# Genetic association between Class II division 1 and division 2 malocclusions with PAX9 (rs8004560) gene polymorphism in a tertiary care hospital

**DOI:** 10.1590/2177-6709.29.6.e2424128.oar

**Published:** 2024-12-16

**Authors:** Monika SHUKLA, Munish REDDY, Kritanjali SINGH, Ruchi SAINI, Pradeep RAGHAV, Kaynat NASSER, Nupur SHARMA, Aastha KAMRANI, Shehla RAFIQUE, Tanjula SHAIR

**Affiliations:** 1 Subharti Dental College, Department of Orthodontics and Dentofacial Orthopedics (Uttar Pradesh, India).; 2 Subharti Medical College & Hospital, Central Research and Incubation Center (Uttar Pradesh, India).; 3 Subharti Medical College, Department of Community Medicine (Uttar Pradesh, India).

**Keywords:** Malocclusion, Genetics, SNP, Má oclusão, Genética, SNP

## Abstract

**Objective:**

To assess whether there is any difference in the genetic association between Class II division 1 (div. 1) and division 2 (div. 2) malocclusions using PAX9 (rs8004560) gene single nucleotide polymorphism (SNP).

**Material and Methods:**

Sixty patients from the Orthodontics department of Subharti Dental College and Hospital (Meerut, India) were divided into two groups: Group 1 (Class II div. 1 malocclusion) and Group 2 (Class II div. 2 malocclusion). Then, 3 mL of blood was collected from each participant. DNA extraction was done, and Sanger Sequencing was performed from extracted DNA samples.

**Results:**

A statistically significant difference was found in the distribution of alleles among Class II div. 1 and Class II div. 2 malocclusions. The homozygous GG allele was the most prevalent among Class II div. 1 patients (76.7%), while the heterozygous AG allele was the most prevalent among Class II div. 2 patients (53.5%). Since GG was the most prevalent allele, it was used as a reference, and AA/AG were compared with GG to confirm the association. The results showed that individuals with the AG genotype seemed to be more susceptible to the development of skeletal Class II div. 2 malocclusion.

**Conclusion:**

The homozygous GG allele was the most prevalent among Class II div. 1 patients, while the heterozygous AG allele was the most prevalent among Class II div. 2 patients, suggesting that there could be a difference between the genetic association of both malocclusions.

## RESUMO

**Objetivo:**

Avaliar se há alguma diferença na associação genética entre as más oclusões de Classe II divisão 1 (div. 1) e divisão 2 (div. 2), usando o polimorfismo de nucleotídeo único (SNP) do gene PAX9 (rs8004560).

**Material e Métodos:**

Sessenta pacientes do departamento de Ortodontia do Subharti Dental College and Hospital (Meerut, Índia) foram divididos em dois grupos: Grupo 1 (má oclusão de Classe II div. 1) e Grupo 2 (má oclusão de Classe II div. 2). Em seguida, 3 mL de sangue foram coletados de cada participante. Foi feita a extração de DNA e foi realizado o sequenciamento Sanger a partir das amostras de DNA extraídas.

**Resultados:**

Uma diferença estatisticamente significativa foi encontrada na distribuição de alelos entre as más oclusões de Classe II div. 1 e Classe II div. 2. O alelo homozigoto GG foi o mais prevalente entre os pacientes Classe II div. 1 (76,7%), enquanto o alelo heterozigoto AG foi o mais prevalente entre os pacientes Classe II div. 2 (53,5%). Como GG foi o alelo mais prevalente, ele foi utilizado como referência, e AA/AG foram comparados com GG para confirmar a associação. Os resultados mostraram que os indivíduos com o genótipo AG pareciam ser mais suscetíveis ao desenvolvimento de má oclusão Classe II div. 2 esquelética.

**Conclusão:**

O alelo homozigoto GG foi o mais prevalente entre os pacientes Classe II div. 1, enquanto o alelo heterozigoto AG foi o mais prevalente entre os pacientes Classe II div. 2, sugerindo que poderia haver uma diferença entre a associação genética de ambas as más oclusões.

## INTRODUCTION

Intricate processes of tissue interactions, cell migrations, and coordinated growth provide a route for the development of the complex craniofacial structures.^1^ Among its three different dimensions, the sagittal one is of utmost importance for orthodontic diagnosis and treatment planning.^2^

The term occlusion was stipulated by Angle, in 1890, as the relationship of the occlusal plane of the teeth of both jaws when they come together. Any deviation from this normal occlusion is termed malocclusion, and is further classified, based on the first molar relationship, into Class I (neutrocclusion), Class II (distocclusion) and Class III (mesiocclusion).^3^ When the maxillary or mandibular bone bases are not in proper proportion, then skeletal Class II or Class III malocclusion can occur, leading to dental distocclusion or mesiocclusion.^4^ Based on upper anterior teeth inclination, this Class II malocclusion can be further divided into two major groups, in which division 1 (div. 1) malocclusions show proclination, and division 2 (div. 2) malocclusions are characterized by retroclination.^5^

Nature or nurture, which one takes charge has been a topic of debate for many years. Whether one’s development is completely predisposed by their “genes” or is mainly influenced by environmental factors is still under investigation.^6^ It is undeniable that genetic modifications play crucial roles, as a robust hereditary pattern of a particular form of skeletal occlusion deformity running in families has been seen among a variety of ethnicities.^7^ Gene modifications that are significant throughout the formation of the craniofacial structure have been linked to the prevalence of the craniofacial abnormalities.^8^

One such gene that plays a crucial role in the formation of the craniofacial region is the paired box 9 (PAX9) gene, which codes for a transcription factor. Humans with cleft lip and palate, palatally impacted canine, oligodontia or hypodontia, and Class II div. 2 malocclusion are often associated with mutant PAX9 genes.^9^ ^,^ ^10^ In addition, it was discovered that the prevalence of dental abnormalities in div. 2 patients were three times higher than in general populations.^11^ In light of this, Wall^12^ investigated whether there was a connection between the Class II div. 2 patients and the hypodontia-associated genes PAX9 or MSX1 (muscle segment homeobox), and proposed that there is a connection between them. 

Recently single nucleotide polymorphisms (SNPs) are in primary focus, as they might affect protein expression and function, to causing a phenotypic change.^13^ The majority of research to date has demonstrated the connection between different gene SNPs and Class III and Class II malocclusions as a whole. However, when considering their subdivisions, there are only a few studies available in the literature.

Although separately both the divisions are found to have a genetic association, still to date there is no comparative study focusing on whether there is any genetic correlation between them. 

Hence, the current study proposed to assess whether there is any genetic association between Class II div. 1 and div. 2 malocclusions using PAX9 (rs8004560) gene SNP, which is suggested to be associated with div. 2 malocclusion.

## MATERIAL AND METHODS

Study participants were taken from the Orthodontics department of Subharti Dental College and Hospital (Meerut, India), based on the exclusion and inclusion criteria described in Table 1. A total of 60 patients with Class II malocclusion (ANB>4º) were selected and further divided into two groups, based on Angle’s classification: Group 1 (Class II division 1 malocclusion), and Group 2 (Class II division 2 malocclusion). 

**Table 1:** Criteria for inclusion and exclusion of patients.

INCLUSION CRITERIA	EXCLUSION CRITERIA
» Subjects’ willingness to participate. » Ages between 12 and 30. » Skeletal Class II pattern (ANB > 4º). » Class II molar and canine relationship (based on Angle’s classification). » Presence of all permanent teeth except third molar. » Patient belonging to the ethnic background of western Uttar Pradesh.	» Patients with previous orthodontic treatment. » Mentally challenged patients. » Patients with any known systemic or endocrinological disorder. » Patients having abnormal oral habits, such as thumb sucking etc.

Prior to study initiation, ethical authorization was obtained from the university ethics committee/medical (UECM, Swami Vivekanand Subharti University, ref nº: SMC/UECM/2022/444/229). Then, a consent was obtained from patients, and 3mL of peripheral blood was drawn from each patient, in EDTA (ethylene diamine tetraacetic acid) tubes. Using extraction kits from QIAGEN, the DNA (deoxyribonucleic acid) from these blood samples was extracted, and it was subjected to Sanger Sequencing. Following, each of the four ddNTPs was tagged with a different fluorescent label, which shows each nucleotide’s fluorescent peak along the template DNA’s length.

**Figure 1:**
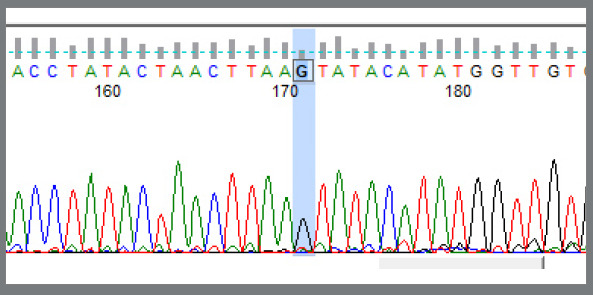
Shows the single black color fluorescent peak at the restriction site: as there is a single peak, it is a homozygous condition. In the fluorescent labeling, the G allele was labeled by black color; then, this figure represents a homozygous condition for G allele, i.e., GG genotype.

**Figure 2:**
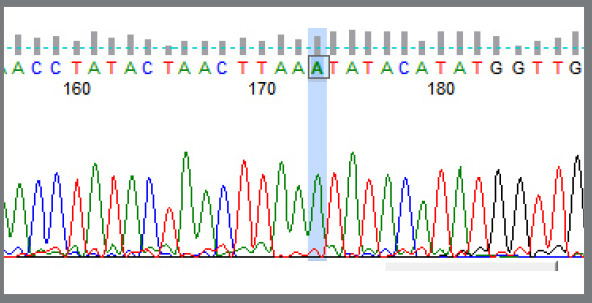
Shows the single green color fluorescent peak at the restriction site: as there is a single peak, it is a homozygous condition. In the fluorescent labeling, the A allele was labeled by green color; then, this figure represents a homozygous condition for A allele, i.e., AA genotype.

**Figure 3:**
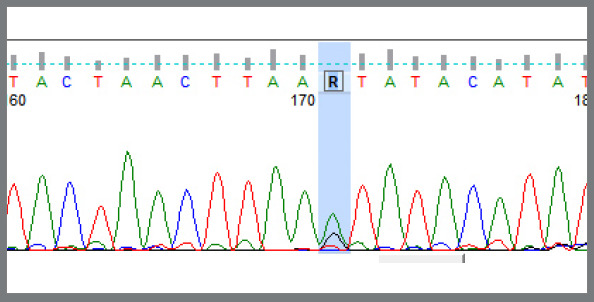
Shows the two fluorescent peaks at the restriction site, one green and one black colour: as there are two peaks, it is a heterozygous condition. In the fluorescent labeling, the A allele was labeled by green colour, and the G allele was labeled by black color; then, this figure represents a heterozygous condition, having both A and G allele, i.e., AG genotype.

In the fluorescent labeling, the G allele was labeled by black color; so, the single black color fluorescent peak at the restriction site represented a homozygous condition for G allele, i.e., GG genotype (Fig 1). The A allele was labeled by green color; so, the single green color fluorescent peak at the restriction site represented a homozygous condition for A allele, i.e., AA genotype (Fig 2). Two fluorescent peaks at the restriction site, one green and one black, represented a heterozygous condition, having both A and G allele, i.e., AG genotype (Fig 3).

## STATISTICAL ANALYSIS

The data was analyzed using the SPSS v. 26.0 software. The level of significance was kept at 5%. The significance of the differences in the observed frequencies of polymorphisms among the groups was assessed by odds ratio (OR) and Fisher Exact test, with a 95% confidence interval (CI). To adjust for the inflated Type I error to be closer to 5%, an adjusted p-value was calculated considering the Benjamini-Hochberg False Discovery Procedure.

## RESULTS

The association of genetic alleles with skeletal Class II div. 1 and div. 2 malocclusions showed that individuals with the AG genotype seemed to be more susceptible for the development of skeletal Class II div. 2 malocclusion (OR: 4.38, 95% CI: 1.42-13.56, *p*=0.0156). Since GG was the most prevalent allele, it was used as a reference, and AA/AG were compared with GG to confirm the association (*p*=0.0156) (Table 2).

**Table 2:** Association of genetic alleles with skeletal Class II div. 1 and div. 2 malocclusions.

Genotype	Div 1	Div 2	OR (85.7%CI)	OR (95% CI)	p-value	Adjusted p-value
GG	23	12	Ref	Ref	Ref	Ref
AG	7	16	4.38 (1.88-10.19)	4.38 (1.42-13.56)	0.0104*	0.0156*
AA	0	2	3096326782	3096326782	0.9999	0.999
AA+AG	7	18	4.93 (2.14-11.36)	4.93 (1.61-15.07)	0.0052*	0.0156*

Binary logistic regression. *Significant association.

On comparative distribution of alleles among skeletal Class II div. 1 and div. 2 malocclusions, it was observed that the homozygous GG allele was the most prevalent among Class II div. 1 patients (76.7%), while the heterozygous AG allele was the most prevalent among Class II div. 2 patients (53.5%), and the difference in the distribution of alleles among skeletal Class II div. 1 and div. 2 malocclusions was significant (Table 3).

**Table 3:** Comparison of the distribution of alleles between skeletal Class II div. 1 and div. 2 malocclusions.

Genotype	Div 1	Div 2	OR (85.7% CI)	p-value	B-H p-value
GG	23	12	Ref	Ref	Ref
AG	7	16	4.38 (1.88-10.19)	0.015	0.0225
AA	0	2	--	0.137	0.999
AA+AG	7	18	4.93 (2.14-11.36)	0.008	0.0225

Fisher exact test.

## DISCUSSION

Development of the craniofacial structures is a complex process and any kind of alterations in it can lead to various malocclusions.^1^ Abundant research has been published concerning the etiology of malocclusions, some suggesting local factors - such as thumb sucking habit, chronic oral ventilation, retained or early loss of deciduous teeth and hormonal imbalance - as causative factors, while a considerable amount of authors still believe that heredity plays a major role and cannot be neglected during diagnosis and treatment planning.^6^

Nakasima et al.^14^ stated that there is a high correlation between parents and their offspring in the Class II and Class Ill groups, thus confirming a strong familial tendency. Thereafter, most of the malocclusion studies focused on SNPs association with Class III and Class II malocclusions as a whole. Considering their subdivision, Markovic^15^ pointed to the genetic origin of div. 2 patients. In addition, few SNP studies have focused on div. 1 and div. 2 malocclusions separately, but a comparative study was still lacking. Hence, the present study was conducted to compare any difference in genetic association between both the divisions and the SNP of the PAX9 gene. 

It was observed that the homozygous GG allele was the most prevalent among Class II div. 1 patients (76.7%), whereas the heterozygous AG allele was the most prevalent among Class II div. 2 patients (53.5%), and the difference in the distribution of alleles among skeletal Class II div. 1 and Class II div. 2 malocclusions was significant (*p* = 0.011).

Hence, it could be stated that, regarding allele distribution, the G allele (non-mutant) of marker rs8004560 was over-represented in the Class II div. 1 patients, while the A allele (minor allele) was over-represented in the Class II div. 2 patients. This suggests that individuals with the A allele seemed to be more susceptible to the development of Class II div. 2 malocclusion. Given that the A allele for rs8004560 is the minor allele and the G allele is the major allele, it can be suggested that the SNP of the PAX9 gene (rs8004560) is related mainly to Class II div. 2 patients, showing that Class II div. 2 is a separate clinical entity, and may be genetically different from Class II div. 1 malocclusion.

The present results are in accordance with the study of Dana Festila et al.^1^, in which four markers were selected from each patient (MyoH1, VDR, PAX9 and RUNX2), and a genetic association was found between the SNP of the PAX9 and this malocclusion (Class II div. 2).

In contrast, the study by Saad et al.^4^ found no correlation between the SNP of the PAX9 (rs8004560) gene and the skeletal Class II malocclusion caused by the retrognathic mandible. However, they also have reported that the reason behind their results may be the small sample size. In addition, the study focused only on Class II malocclusion, and did not assess their subdivisions. 

The present findings are consistent with the study by Morford et al.^16^, which found a hereditary correlation between Class II div. 2 individuals and SNP of the PAX9 gene, as a borderline association of all Class II div. 2 subjects with PAX9 (rs8004560) was identified. The study by Wall^12^ investigated whether there was a connection between the Class II div. 2 individuals and the hypodontia-associated genes PAX9 or MSX1, and proposed that there was a connection between the SNP PAX9 rs8004560 and Class II div. 2 individuals. This outcome also validates the findings of the present study.

Although the difference in the distribution of alleles among skeletal Class II div. 1 and Class II div. 2 malocclusions was significant and a major number of individuals in the Class II div. 2 group showed SNP of the PAX9 gene, there were still a few individuals in the Class II div. 2 group who showed negative results, and some Class II div. 1 individuals also showed the SNP of PAX9 gene. This suggested that either this phenotypic representation was modified by the involvement of some other set of genes or any epigenetic factors. Henceforth, genetics should not be considered as the only causative factor, and the epigenetic factors should be taken into due consideration. 

## LIMITATIONS OF THE STUDY

The present study have considered only one gene (PAX9), but malocclusions may have multifactorial etiology. Thus, other genes may also be considered in further studies (especially those that are related with tooth agenesis), to validate whether there is any association of genes involved in tooth agenesis and Class II div. 2 malocclusion.

The present study have only assessed the SNP of the patients with malocclusion, but, to confirm the genetic association, inheritance patterns (maternal/paternal) should be considered. So, future studies could include parents in the sample.

## CONCLUSION

A statistically significant difference in the distribution of alleles was observed between skeletal Class II div. 1 and div. 2 malocclusions, in which the homozygous GG allele was commonly observed among Class II div. 1 patients (76.7%), while the heterozygous AG allele was commonly observed among Class II div. 2 patients (53.3%). It was also found that the homozygous AA allele was seen only in 6.7% of Class II div. 2 patients. 

This allelic distribution implies that the minor A allele was over-represented in Class II div. 2 patients (60%), hence it might be suggested that the SNP of the PAX9 gene is associated with Class II div. 2 malocclusion.

The present study also reported that 40% of Class II div. 2 individuals had no minor allele, and 23.3% of Class II div. 1 individuals had a minor allele, but lacked the Class II div. 2 clinical features. This implied that there might be an involvement of another gene set, variation in gene expression, or any epigenetic factor that altered the phenotypic representation.
